# Silent Myocardial Ischemia: From Pathophysiology to Diagnosis and Treatment

**DOI:** 10.3390/biomedicines12020259

**Published:** 2024-01-23

**Authors:** Panagiotis Theofilis, Alexios S. Antonopoulos, Marios Sagris, Aggelos Papanikolaou, Evangelos Oikonomou, Konstantinos Tsioufis, Dimitris Tousoulis

**Affiliations:** 1 1st Department of Cardiology, “Hippokration” General Hospital, National and Kapodistrian University of Athens, 11527 Athens, Greece; panos.theofilis@hotmail.com (P.T.); antonopoulosal@yahoo.gr (A.S.A.); masagris1919@gmail.com (M.S.); agepap25@otenet.gr (A.P.); ktsioufis@gmail.com (K.T.); 2 3rd Department of Cardiology, Thoracic Diseases General Hospital “Sotiria”, National and Kapodistrian University of Athens, 11527 Athens, Greece; boikono@gmail.com

**Keywords:** silent ischemia, coronary artery disease, pain

## Abstract

Silent myocardial ischemia (SMI), characterized by a lack of overt symptoms despite an inadequate blood supply to the myocardium, remains a challenging entity in cardiovascular medicine. The pathogenesis involves intricate interactions of vascular, neurohormonal, and metabolic factors, contributing to perfusion deficits without the characteristic chest pain. Understanding these mechanisms is pivotal for recognizing diverse clinical presentations and designing targeted interventions. Diagnostic strategies for SMI have evolved from traditional electrocardiography to advanced imaging modalities, including stress echocardiography, single-photon emission computed tomography (SPECT), positron emission tomography (PET), and cardiac magnetic resonance imaging (MRI). Treating SMI is a matter of ongoing debate, as the available evidence on the role of invasive versus medical management is controversial. This comprehensive review synthesizes current knowledge of silent myocardial ischemia, addressing its pathophysiology, diagnostic modalities, and therapeutic interventions.

## 1. Introduction

Coronary artery disease (CAD) remains the leading cause of morbidity and mortality worldwide [1], despite the technological advancements assisting doctors in its prompt recognition and management. Its cardinal symptom is angina pectoris, which can be treated pharmacologically or invasively [2]. Although the majority of patients suffering from CAD report angina or equivalents, there is a significant proportion exhibiting ischemia without overt symptomatology, also known as silent myocardial ischemia (SMI). This condition poses a significant challenge in contemporary cardiovascular medicine. This ischemic phenomenon occurs when there is an inadequate supply of oxygen-rich blood to the heart, usually due to narrowed or blocked coronary arteries. Unlike typical myocardial ischemia, which presents with chest pain (angina) or discomfort, SMI does not produce these recognizable warning signs, making its diagnosis and management complex [3]. The insidious progression of SMI poses a substantial burden on individuals and healthcare systems, contributing to the burden of cardiovascular morbidity and mortality. It often remains undetected until more severe complications, such as heart attacks or irreversible heart damage, occur. Consequently, addressing SMI involves a multidimensional approach focusing on early detection, risk factor management, and preventive strategies to mitigate its impact on cardiovascular health.

This review endeavors to unravel the multifaceted aspects of SMI, spanning from the intricate pathophysiological underpinnings to the evolving landscape of diagnostic modalities and therapeutic interventions.

## 2. Mechanisms of Ischemic Cardiac Pain

**Figure 1:**
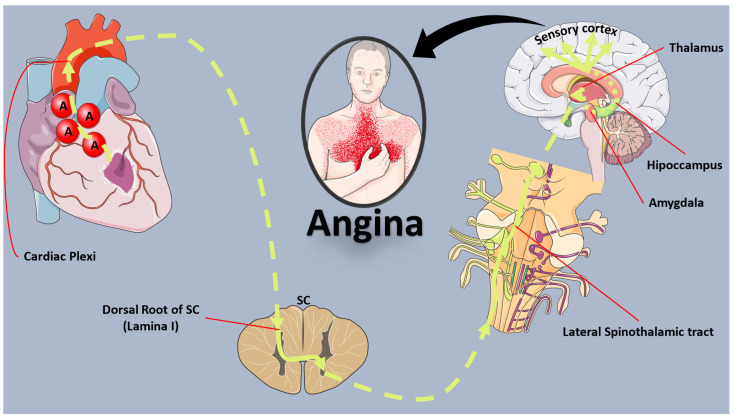
Schematic representation of the mechanisms of angina. A: adenosine; SC: spinal cord.

Visceral pain is orchestrated through the autonomic nervous system, although investigating human autonomic function poses challenges, often necessitating invasive methods, thereby constraining our comprehension. The heart receives extensive autonomic innervation, with well-recognized efferent stimulation effects [4]. Vagal stimulation decreases heart rate and has a negative inotropic impact, while sympathetic stimulation elicits opposing responses. In the context of cardiac pain, it appears that afferent sympathetic nerve activity, principally induced by adenosine, is pivotal for conveying pain signals from the heart to the spinal cord and brain (Figure 1), overshadowing the minor role of the vagus nerve in afferent pain transmission [5].

Afferent sympathetic neurons traverse from the myocardium to the superior or inferior cardiac plexus, proceeding without synapsing through the sympathetic ganglion chain to the dorsal horn of the spinal cord [5]. This connectivity, particularly to lamina I neurons, suggests a convergence point for somatic and visceral afferent pathways, possibly giving rise to the referred pain characteristic of angina pectoris. The extensive connections between the cardiac sympathetic plexi, the sympathetic ganglion chain, and the spinal cord contribute to the potential expression of angina across a significant portion of the upper body.

Moreover, afferent neuronal connections from the tracheo-bronchial tree, lungs, esophagus, and stomach converge with cardiac afferent fibers at the same segmental levels of the spinal cord [5]. In the dorsal root entry zone’s lamina I, afferent autonomic neurons may extend connecting branches rostrally and caudally, potentially synapsing onto a transmitter cell which receives inputs from sensory nociceptive neurons [5]. This intricate network suggests a common connection in lamina I as the point where ‘angina’ information gains access to recognized rostral pain pathways. However, the precise mechanisms and potential crosstalk remain speculative.

Alterations in inhibitory and excitatory neurotransmitters at the dorsal root entry zone may explain altered cutaneous sensitivity within the patient’s described “angina territory” [5]. Following stimulation of neurons in the lateral spinothalamic tract, pain processing involves brain areas such as the peri-aqueductal grey matter, nucleus raphe magnus, insula, thalamus, amygdalo-hippocampal apparatus, sensory cortex, and frontal cortex, culminating in the conscious perception of pain.

Distinctive features of angina, shared with other visceral pain syndromes, differentiate it from somatic pain, primarily characterized by its poorly localized nature [5]. The convergence of visceral autonomic inputs onto common transmitter cells, shared by both visceral and somatic afferents, contributes to this phenomenon. Furthermore, individual variations in the character, intensity, and location of angina perception arise due to the failure of these common spinal routes to categorize autonomic information as nociceptive. Patients often refrain from characterizing angina as painful, opting for terms like “discomfort”, “pressure”, or “heaviness”.

Traditional functional brain area classifications may provide limited assistance in understanding the neurobiology of angina, given emerging evidence of the motor cortex’s role in maintaining chronic pain states [5]. Associations between angina pectoris and myalgic pain in certain muscle groups underscore the intricate relationship between visceral and musculoskeletal pain. Despite limited knowledge of higher brain centers’ processing of angina signals, studies on autonomic responses to esophageal pain offer parallels, suggesting a neuroticism-dependent range of responses. This nuanced interplay underscores the complexity of angina neurobiology, an area ripe for further exploration and development.

## 3. Mechanisms of Silent Myocardial Ischemia

SMI is characterized by objective evidence of myocardial ischemia in the absence of angina or its equivalents, such as dyspnea, nausea, and diaphoresis, in patients with CAD [6]. This condition results from an imbalance between the consumption and production of adenosine triphosphate (ATP), leading to biochemical events without the manifestation of typical symptoms. Notably, a substantial percentage of transient ischemic episodes, ranging from 50% to 70%, do not present with anginal chest pain [7]. The absence of pain in SMI can lead to increased morbidity and mortality due to the delayed recognition of ischemic events. SMI is categorized into three types [8], as shown in Table 1.

Mechanistically, SMI involves the occurrence of myocardial ischemia without angina or its equivalents. Spinal cardiac fibers transmit anginal pain through afferent pathways to the thalamus and, subsequently, to the cerebral cortex. Biochemical substances and receptors, including substance P, glutamate, and transient receptor potential vanilloid-1 (TRPV1) receptors, play crucial roles in the neurotransmission of cardiac pain [9]. Tumor necrosis factor α (TNF-α) or Interleukin-1β (IL-1β) can enhance pain transmission signals by lowering the activation threshold of nociceptors [9].

Vagal cardiac afferent fibers may contribute to SMI through the nucleus of the solitary tract and the C1–C2 spinal segments, mediating both typical and atypical anginal pain [9]. Sensory input from other visceral organs can mimic cardiac pain due to convergence with cardiac input onto spinothalamic tract neurons, thereby reducing cardiac pain. Additionally, the psychological state of an individual and the descending pathways from the nucleus raphe magnus and the pons can modulate cardiac nociception.

The ischemic burden, reflecting the combined presence of overt and silent ischemia, correlates with the magnitude, duration, and severity of ischemic episodes. Two critical parameters in SMI genesis are the magnitude of the ischemic stimulus and the pain threshold. The pain threshold may be elevated, leading to a lack of pain sensation, as seen in conditions such as cardiovascular autonomic neuropathy, particularly in diabetic patients [10]. Studies utilizing meta-iodobenzylguanidine have demonstrated a notably diminished myocardial sympathetic innervation in individuals with diabetes compared to those without diabetes [11]. Moreover, there is evidence of a diffuse abnormality in meta-iodobenzylguanidine uptake among diabetic patients with silent myocardial ischemia, indicative of sympathetic denervation. Autopsy studies further support these findings, revealing in individuals with diabetes a fragmentation of afferent sympathetic fibers in the myocardium, a reduced number of fibers, and beaded thickening of nerves [12]. These observations align with characteristics of autonomic sensory neuropathy, offering a potential explanation for the occurrence of silent ischemia in diabetes. In summary, the etiology of silent ischemia in the context of diabetes appears to involve anatomical disruptions in cardiac sensory nerve fibers.

## 4. Epidemiology and Prognosis of Silent Myocardial Ischemia and Infarction

Estimating the prevalence of SMI is challenging due to its silent nature, but it is believed to affect 2–4% of the general population, with higher prevalence in certain patient groups, such as those with stable angina or diabetes. When considering silent MI prevalence in the general population, it ranges from 0.5% (in younger individuals) to 6.4% (in the elderly) [13]. SMI exhibits a circadian pattern, with a higher incidence in the morning, possibly linked to physiopathologic changes during this period, including increased heart rate, blood pressure, and catecholamine levels, enhanced platelet aggregation, and decreased fibrinolytic activity [14]. The rates of SMI according to sex are contradictory across the existing studies. Women, who often present atypical symptoms [15], were found to be independently associated with the presence of SMI in one study [16]. However, other studies have shown that SMI prevalence is higher in males compared to females [17,18]. As far as racial disparities are concerned, black patients have been found to be nonsignificantly related to a higher incidence of SMI compared to white patients [18].

Patients with type 2 diabetes are at a heightened risk of developing SMI, contributing to worse long-term outcomes in female patients particularly [19]. In a recent analysis of the ACCORD (Action to Control Cardiovascular Risk in Diabetes) study involving individuals with type 2 diabetes mellitus, further evidence was provided concerning the association of cardiac autonomic neuropathy (CAN) and SMI [20]. At the baseline within the analyzed cohort, the prevalence of CAN was 18.6%. Those with diminished heart rate variability exhibited higher values for body mass index, HbA1C, and diabetes duration and lower high-density lipoprotein cholesterol and estimated glomerular filtration rate. Additionally, this group was more likely to include current smokers, insulin users, or individuals with a history of retinopathy. Over a median follow-up period of 4.9 years, individuals with CAN demonstrated a silent myocardial infarction (MI) incidence rate exceeding 1.5 times that of those without CAN. Upon a multivariable adjustment, CAN exhibited a significant association with an elevated risk of silent myocardial infarction (HR 1.91 [95% CI 1.14–3.18]). The diagnostic performance metrics of CAN for identifying silent myocardial infarction were as follows: sensitivity 30.1%, specificity 81.5%, positive predictive value 2.4%, and negative predictive value 98.7%. In the group of patients with diabetes mellitus, the presence of chronic kidney disease (CKD) might be another factor favoring the development of SMI. Honda et al. conducted a study of 461 patients with diabetes mellitus, free of clinical cardiovascular disease, who subsequently underwent an ergometer exercise test followed by invasive coronary angiography in case of a positive result or failure to achieve 90% of the targeted heart rate [21]. The prevalence of SMI in this cohort was approximately 18%. SMI demonstrated a higher prevalence among individuals with CKD, and its incidence correlated with the stage of CKD in the asymptomatic patients with diabetes mellitus. Furthermore, the patients with both CKD and SMI experienced unfavorable clinical outcomes.

Perioperative SMI deserves an honorable mention as it is relatively common. In the Perioperative Ischemic Evaluation (POISE)-1 trial, 5.0% of patients experienced perioperative MI, with 65% of these individuals not presenting ischemic symptoms [22]. The thirty-day mortality rates were comparable between the patients with perioperative MI, whether accompanied by ischemic symptoms or not. Puelacher et al. conducted troponin screening in a prospective cohort of 2018 patients undergoing noncardiac surgery, revealing that only 6% of those with perioperative MI reported typical chest pain [23]. Their findings indicated similar 30-day mortality rates between patients with perioperative MI lacking other ischemic MI criteria and those exhibiting at least one ischemic feature. However, the issue of prognostically significant myocardial injury following noncardiac surgery extends beyond perioperative MI. The Vascular Events in Noncardiac Surgery Patients Cohort Evaluation Study (VISION) involved a representative sample of over 40,000 patients aged 45 and older. In VISION, 5191 patients encountered myocardial injury after noncardiac surgery, characterized by troponin elevation attributable to an ischemic cause [24]. The study revealed that both perioperative troponin elevation without meeting the universal definition of MI and perioperative MI fulfilling the universal definition were associated with 30-day mortality after noncardiac surgery. Remarkably, 93% of patients with myocardial injury after noncardiac surgery in VISION were asymptomatic, and 22% met the universal definition of MI. Finally, in the study of Wilcox et al., the prevalence of postoperative MI was 0.37%, with affected individuals facing an augmented short-term risk of death [25]. Among the identified independent risk factors were smoking exposure and diabetes mellitus.

Prognostically, a silent MI might also signify an increased risk of sudden cardiac death (SCD), as reported by an analysis of the ARIC (Atherosclerosis Risk in Communities) study and the CHS (Cardiovascular Health Study) [26]. In particular, the combined hazard ratios for silent and clinical MI in relation to sudden cardiac death (SCD) were 2.65 (95% CI: 2.18–3.23) and 3.99 (95% CI: 3.34–4.77), respectively. The risk of SCD associated with silent MI is more pronounced in white individuals, males, and those of younger age. The population-attributable fraction of SCD attributed to silent MI was 11.1%, and silent MI was linked to an absolute risk increase of 8.9 sudden cardiac deaths per 1000 person-years. Additionally, the inclusion of silent MI significantly enhanced the predictive accuracy for both SCD and non-SCD.

While the evidence surrounding the prognostic importance of silent MI is mounting, a recent study has questioned this hypothesis. Based on an analysis of the multi-ethnic study of atherosclerosis involving intermediate-risk participants free from clinical cardiovascular disease, the investigators initially found that silent MI (prevalence 2.2%) was associated with a greater incidence of atherosclerotic cardiovascular disease [27]. However, when this was added on top of the pooled cohort equation for incident events, there was only a modest, nonsignificant improvement in discrimination and reclassification.

## 5. Screening and Diagnosis of Silent Ischemia

In this section, we describe the array of diagnostic methods available to detect SMI (Table 2). As the majority of individuals presenting with SMI are affected by CAN in the setting of DM, most studies involve participants from this subgroup of patients. While screening and diagnosing SMI might seem attractive, the latest European Society of Cardiology (ESC) guidelines on chronic coronary syndromes advise against the use of functional imaging in the general population of asymptomatic subjects, providing only a weak recommendation (IIb-C) for those at the highest risk (presence of DM, strong family history of CAD, very high risk of having CAD based on other tests) [28].

### 5.1. Exercise Treadmill Test

An exercise treadmill test (ETT), also known as a stress test or treadmill stress test, is a diagnostic procedure performed to assess the cardiovascular system’s response to physical exertion. This test is commonly used to evaluate the presence of CAD, determine exercise capacity, and identify abnormal heart rhythms. Findings such as ST-segment changes on the ECG during exercise or a hypotensive response are considered abnormal.

An ETT may sometimes be ordered as the first non-invasive method of ischemia assessment even in asymptomatic individuals. Among the earliest studies using this approach was the one conducted by Laukkanen et al. in 1769 middle-aged male individuals who were free from cardiovascular disease [29]. SMI during exercise was present in 10.7% of the participants, among whom 15.3% faced an acute coronary event and 7.9% died from cardiovascular causes. After an adjustment for traditional cardiovascular risk factors, the presence of SMI during exercise was accompanied by a 1.7-fold higher risk of acute coronary events and a 3.5-fold higher risk of cardiovascular mortality, with those findings being of greater magnitude in individuals with coexistent smoking, hyperlipidemia, or hypertension. Critically, this study highlighted that the ST-segment changes that are prolonged or develop after exercise are also predictive of adverse outcomes.

### 5.2. Echocardiography

Echocardiography plays a crucial role in the assessment of SMI as it provides valuable information about cardiac structure and function, helping clinicians identify areas of compromised blood flow and potential ischemic damage. TTE is the most commonly used echocardiographic technique for SMI assessment. Echocardiographic images are carefully analyzed for regional wall motion abnormalities and changes in ejection fraction. Strain imaging is a technique that assesses the deformation of myocardial tissue during the cardiac cycle. Reduced strain in specific regions may indicate compromised blood flow and myocardial ischemia, even in the absence of symptoms. Recently, Albenque et al. studied the importance of left ventricular global longitudinal strain (LV GLS) in predicting positive stress echocardiography in asymptomatic patients with diabetes mellitus and high-risk features [30]. The investigators observed a potent association between an impaired LV GLS and a positive stress echocardiography, indicative of SMI. When this parameter was added to the multivariable model, it significantly enhanced its predictive power. As the sample size of this study was small, further evidence in this field is required to validate these findings.

Stress echocardiography involves inducing stress, either through exercise or pharmacological agents, to evaluate the heart’s response under increased demand. During stress, echocardiographic images are obtained to assess changes in wall motion, which can indicate areas of reduced blood supply to the heart [42]. Dobutamine, a medication that stimulates the heart, may be used when physical exercise is not feasible. Dobutamine stress echocardiography helps mimic the effects of exercise on the heart and assess regional wall motion abnormalities [42]. In 161 asymptomatic patients with type 2 diabetes mellitus and no prior CAD history, abnormalities in dobutamine stress echocardiography (DSE), present in 28% of the studies, were predictive of future adverse cardiovascular events during a median follow-up of 5 years, adding incremental prognostic value to the clinical variables [31]. Similarly, SMI on exercise or dobutamine stress echocardiography was predictive of incident major adverse cardiac and cerebrovascular events in another study of asymptomatic patients with type 2 diabetes mellitus [32]. Other than inducible ischemia on SE, coronary microvascular dysfunction determined by an abnormal coronary flow reserve might serve as another element of greater cardiovascular risk in asymptomatic, high-risk patients with DM [33]. It should be stressed that, according to a study conducted among patients with comparable degrees of ischemia in DSE, those having SMI compared to symptomatic CAD faced a higher risk of death or MI, possibly due to being undertreated [43]. Compared to the other popular approach—single-photon emission computed tomography (SPECT)—a randomized study by Jacqueminet et al. in 204 asymptomatic, high-risk patients with type 2 diabetes mellitus found a similar detecting capability of SE towards SMI and a similar prognostic power [44]. Apart from wall motion abnormality assessment during an SE, other indices may be indicative of SMI and CAD, such as the E/e’ ratio before and after exercise, as documented by Paraskevaidis et al. in 105 patients with diabetes mellitus [45].

### 5.3. Cardiac MRI

Cardiac magnetic resonance imaging (MRI) stands as a powerful non-invasive imaging modality for the detection and characterization of SMI, as it employs various sequences to capture high-resolution images of the heart. One commonly used sequence is myocardial perfusion imaging, utilizing gadolinium-based contrast agents. Following intravenous administration, these agents highlight regions of the myocardium with compromised blood flow during stress and rest conditions. Perfusion abnormalities, manifested as subendocardial hypoenhancements, become apparent on the images, allowing for the identification of ischemic territories [46]. Additionally, cine MRI sequences enable the assessment of myocardial function by providing dynamic images of the cardiac chambers and myocardial wall motion. The evaluation of regional wall motion abnormalities during stress and rest can further aid doctors in the identification of areas susceptible to ischemia [47]. The quantitative analysis of myocardial strain and ejection fraction enhances the sensitivity of cardiac MRI in detecting subtle functional abnormalities associated with silent myocardial ischemia. Late gadolinium enhancement (LGE) imaging is another crucial aspect of cardiac MRI for ischemic heart disease evaluation. LGE highlights areas of myocardial scarring or fibrosis, which may result from prior ischemic events [47]. Distinguishing between reversible perfusion defects and irreversible scar tissue is essential for risk stratification and therapeutic decision-making [47]. Cardiac MRI is also capable of assessing myocardial viability through techniques such as T1 and T2 mapping [47]. These sequences provide insights into tissue characteristics, allowing clinicians to differentiate between viable myocardium and irreversibly damaged tissue. Moreover, stress perfusion MRI, often induced pharmacologically, allows for the evaluation of dynamic changes in myocardial perfusion during increased cardiac demand, aiding in the identification of ischemic regions [47].

Recent studies have examined the role of cardiac MRI in SMI. In 327 individuals at increased risk of cardiovascular events (but free from symptoms/clinical CAD), dobutamine stress-induced myocardial ischemia on cardiac MRI was predictive of future cardiovascular events and survival, especially in men [34]. In a retrospective cohort study of 903 asymptomatic individuals with ≥2 risk factors but without known CAD, dipyridamole stress cardiac MRI revealed SMI in approximately 12% of them [35]. At the same time, an unrecognized MI was evident in 10.6% of patients, among whom 32.3% had SMI. Following the performance of cardiac MRI, 62.7% of patients with SMI underwent coronary revascularization within 90 days. During a median 9.2-year follow-up, SMI and unrecognized MI were independent predictors of incident major adverse cardiovascular events (MACE) and cardiovascular mortality. Interestingly, early revascularization following cardiac MRI was not accompanied by a protective long-term effect with regards to MACE.

### 5.4. Single-Photon Emission Computed Tomography

SPECT is a non-invasive nuclear imaging technique widely employed in the field of cardiology for the detection of silent myocardial ischemia. It involves the administration of a radiopharmaceutical, typically Technetium-99m (Tc-99m), that emits gamma rays. The radiopharmaceutical is preferentially taken up by myocardial tissue in proportion to regional blood flow. Following administration, a gamma camera rotates around the patient, capturing the emitted gamma rays from various angles. The acquired data are then processed to generate tomographic images, providing a three-dimensional representation of myocardial perfusion. In the context of silent myocardial ischemia, SPECT serves as a valuable diagnostic tool by assessing regional myocardial perfusion defects. Areas of the myocardium with compromised blood flow will exhibit reduced radiotracer uptake, leading to identifiable perfusion abnormalities on the SPECT images [48]. These perfusion defects may be indicative of underlying coronary artery disease or myocardial ischemia. The quantitative analysis of SPECT images allows for the determination of the severity and extent of myocardial perfusion abnormalities [48]. Additionally, stress and rest imaging protocols are commonly employed to evaluate myocardial perfusion under both physiological and resting conditions. By comparing stress and rest images, clinicians can identify reversible perfusion defects, indicative of ischemia, as opposed to fixed defects, suggestive of scar tissue from prior infarction [48].

In the Detection of Ischemia in Asymptomatic Diabetics (DIAD) study performed in the early 2000’s, adenosine Tc-99m sestamibi SPECT revealed SMI in over 20% of asymptomatic individuals with DM, with moderate or large perfusion defects being present in 6% of the tested subjects [36]. It should be noted that ischemic ST-segment depression in the absence of perfusion defects was identified in 4% of the patients, predominantly in women. Hypertensive individuals free from CAD also exhibited SMI at a high rate (28%), as observed in a study utilizing Dipyridamole Tc-99m sestamibi SPECT [37]. In a large-scale study of 3664 patients undergoing exercise Tc-99m sestamibi SPECT, the prevalence of SMI was approximately 21%, with 6% of patients having high-risk ischemia defined as ischemia in ≥7.5% of the myocardium [38]. Concerning the prognostic significance of SPECT, a summed stress score ≥9 was an independent predictor of incident MACE in asymptomatic patients with type 2 DM [39]. In more contemporary cohort studies, such as the one performed by Xiao-Rong et al. in 614 asymptomatic patients with type 2 DM, the prevalence of SMI based on an adenosine Tc-99m sestamibi SPECT was estimated at 21.3%, being higher in male subjects, in those with diabetic retinopathy, or in the setting of increased low-density lipoprotein cholesterol levels [40].

Apart from SMI, the role of SPECT in patients with silent MI has been investigated. According to the study of Ammar et al., patients with unrecognized MIs presented with smaller infarct size and lower degrees of ischemia compared to subjects with clinically recognized MIs [49]. However, the prognosis of both patient subgroups was similar, even if cases of 0% infarct size with only ECG abnormalities were indicative of unrecognized MIs.

### 5.5. Positron Emission Tomography

Positron emission tomography (PET) is a sophisticated nuclear imaging modality utilized in cardiology that offers superior spatial resolution and quantitative capabilities, making it a valuable tool in the assessment of myocardial perfusion and metabolism. In the context of silent myocardial ischemia, PET imaging involves the administration of a positron-emitting radiopharmaceutical, commonly Rubidium-82 or Nitrogen-13 ammonia [48]. These radiotracers are preferentially taken up by myocardial tissue in proportion to regional blood flow. Positrons emitted during radioactive decay combine with electrons, resulting in the emission of two annihilation photons in opposite directions. Detectors surrounding the patient capture these photons, allowing for the precise localization of positron-emitting sources within the myocardium.

PET provides high-resolution three-dimensional images of myocardial perfusion, allowing for the identification and quantification of perfusion abnormalities associated with silent myocardial ischemia. By evaluating the distribution of radiotracer uptake, clinicians can discern regions of the myocardium with compromised blood flow [48]. Moreover, PET’s quantitative capabilities enable the calculation of myocardial blood flow values, aiding in the assessment of perfusion at a more granular level [48]. Dynamic PET imaging, often performed during stress and rest conditions, enables the evaluation of myocardial perfusion under different physiological states. Stress conditions may be induced pharmacologically or through exercise, simulating increased cardiac demand. Subsequent rest imaging allows for the identification of reversible perfusion defects, indicative of ischemia, versus fixed defects, suggestive of scar tissue from previous infarction [48].

The role of PET in SMI has been scarcely investigated to date. In a large study of 2730 individuals with DM (23.7% asymptomatic) undergoing rubidium-82 rest/stress PET myocardial perfusion imaging, the presence of SMI was evidenced in approximately 30.5%, being significant in 12.5% of patients [41]. However, based on subsequent analyses, reduced myocardial blood flow reserve (MBFR), and not SMI or symptoms, was predictive of incident all-cause mortality. Reduced MBFR was found in 62% of asymptomatic patients.

### 5.6. Silent Myocardial Ischemia Screening after Revascularization

SMI might be a prominent feature in patients after coronary revascularization. In the study of Georgoulias et al., 246 consecutive patients underwent Tc-99m tetrofosmin SPECT 5–7 months after percutaneous coronary intervention (PCI). The summed stress score and summed difference score derived from the SPECT evaluation were predictive of incident MACE (cardiac mortality, nonfatal MI, late revascularization) during a median follow-up of 9.5 years [50]. However, the role of repeat revascularization remains to be further elucidated in this setting, since it did not provide any additional survival benefit in cases of positive-for-ischemia stress echocardiography [51]. This observation was also documented in a greater cohort (6750 patients), utilizing another functional testing modality (SPECT) [52]. Moreover, the positivity of the diagnostic test mostly concerns remote vessel areas that are prone to progressive atherosclerosis rather than restenosis of a previously revascularized lesion [53]. Based on the available evidence, screening asymptomatic subjects after coronary revascularization appears to be of low value in terms of the repeat revascularization requirement and should generally be avoided.

## 6. Management of Silent Myocardial Ischemia

The identification of SMI might have therapeutic implications. The use of anti-ischemic medication and aspirin might benefit patients with SMI, as shown in a small-scale randomized trial with a mean follow-up of 11.2 years showing a reduction in MACE compared to only controlling risk factors [54]. The intensification of medical treatment might lead to the resolution of SMI according to the findings of the DIAD study [55].

While optimal medical therapy is widely recommended for these patients, the performance of PCI is a matter of ongoing debate. In a post hoc analysis of the COURAGE trial, the performance of PCI on top of optimal medical therapy in the setting of SMI did not provide additional benefits [56]. Other studies, although nonrandomized, have also proven that PCI does not improve clinical outcomes. Koshy et al. found that asymptomatic patients undergoing PCI had a worse periprocedural and long-term prognosis compared to symptomatic individuals [57]. The investigators stressed the possible role of older age and a greater comorbidity burden as important contributing factors. However, asymptomatic status was an independent predictor of all-cause mortality after a multivariate analysis (adjusted HR: 1.39, 95% CI 1.16–1.66, *p* < 0.001). Moreover, it is unclear what percentage of patients truly had SMI, as no data on previous functional diagnostic tests are available. On the contrary, Choi et al., through an analysis of 1473 asymptomatic patients with SMI, found a significantly lower risk of mortality with revascularization compared to medical treatment alone even after adjustment for multiple confounders [58]. Importantly, the resolution of ischemia following revascularization was related to a favorable prognosis. Last but not least, a post hoc analysis of asymptomatic patients participating in the ISCHEMIA trial could provide further answers to the existing evidence gaps.

According to the latest guidelines by the ESC [59], myocardial revascularization in the setting of SMI is indicated to improve prognosis in left main disease with stenosis >50%, proximal LAD stenosis >50%, two- or three-vessel disease with stenosis >50% and left ventricular ejection fraction ≤35%, large left ventricular ischemic area (>10%), abnormal invasive fractional flow reserve, or single remaining patent coronary artery with stenosis >50%. Based on the ESC guidelines on chronic coronary syndromes, revascularization ought to be considered in cases of SMI with a large ischemic area (>10% of the left ventricle) in stress testing or in the presence of significant stenoses (>90% diameter stenosis, fractional flow reserve ≤0.80 in major vessel, significant left ventricular dysfunction with left ventricular ejection fraction ≤35% due to CAD) [28]. As far as the latest ACC/AHA/SCAI guidelines for coronary artery revascularization are concerned, significant left main disease, multivessel CAD with left ventricular systolic dysfunction, or three-vessel disease with normal left ventricular systolic function are the main indications that revascularization could improve the prognosis [60]. At the same time, the survival benefit of revascularization is uncertain in cases of proximal LAD significant stenosis and a normal left ventricular ejection fraction, while it is not recommended in cases of one- or two-vessel CAD not involving the proximal LAD or in patients who have one or more arteries which are anatomically or functionally nonsignificant [60].

## 7. Conclusions

Silent myocardial ischemia represents a challenging entity in everyday clinical practice due to its asymptomatic nature. Its reasonably high prevalence (~20%) and prognostic implications require prompt recognition and management. Despite the availability of functional tests to aid its diagnosis, the field of interventional treatment remains obscure as no convincing evidence has been provided to date on the beneficial effect of myocardial revascularization.

## Figures and Tables

**Table 1:** Classification of silent myocardial ischemia [8].

Type	Definition
I	Occurring in patients with asymptomatic coronary artery disease without collateral anginal symptoms.
II	Manifesting in patients with a history of myocardial infarction.
III	Observed in patients with concurrent or collateral manifestations of chronic stable angina, unstable angina, and vasospastic angina.

**Table 2:** Overview of studies utilizing various methods of silent myocardial ischemia assessment.

Author	Year	Population (Number of Participants)	Method	Findings
Laukkanen et al. [29]	2001	Men with no prior CAD (1769)	ETT	Prevalence of SMI during exercise: 15.3% SMI during exercise → 1.7-fold risk of ACS and 3.5-fold risk of CAD mortality
Albenque et al. [30]	2022	Asymptomatic, with DM and high-risk features (273)	TTE	OR 1.39 (95% CI 1.14–1.70) per percentage LV GLS increase for predictive positive stress echocardiography OR 5.16 (95% CI 1.96–13.59) for LV GLS worse than −18% for predictive positive stress echocardiography
Sozzi et al. [31]	2007	Asymptomatic, with DM and no prior CAD (161)	DSE	Ischemia prevalence: 28% Adverse events were higher in the presence of an abnormal DSE (24% vs. 11% at 5 years)
Fateh-Moghadam et al. [32]	2009	Asymptomatic, with type 2 DM (211)	ESE or DSE	SMI: 28.9% Positive SE associated with a 13.5-fold risk of MACCE.
Cortigiani et al. [33]	2017	Asymptomatic, high-risk patients with DM (230)	SE + LAD-CFVR	Ischemia or abnormal LAD-CFVR in 23% and was predictive of incident MACE (HR 6.12, 95% CI 3.22–11.62)
Stacey et al. [34]	2018	Asymptomatic, high-risk patients (327)	DS-cardiac MRI	SMI was predictive of future cardiovascular events/survival (adjusted HR: 4.07, 95% CI 1.95–8.73, *p* < 0.001)
Pezel et al. [35]	2021	Asymptomatic, high-risk patients (903)	Dipyridamole cardiac MRI	SMI: 12.2% SMI was an independent predictor of incident MACE (HR 6.66, 95% CI 4.41–9.23) and cardiovascular mortality (HR: 6.21, 95% CI 3.89–9.48)
DIAD [36]	2004	Asymptomatic, with type 2 DM (522)	Adenosine Tc-99m sestamibi SPECT	SMI: 22% Moderate/Large defects: 6%
Lacourciere et al. [37]	2006	Asymptomatic, with essential hypertension (543)	Dipyridamole Tc-99m sestamibi SPECT	Abnormal SSS: 28% Greater prevalence and severity of SMI in coexisting DM
Zellweger et al. [38]	2009	Asymptomatic, with no prior CAD (3664)	Exercise Tc-99m sestamibi SPECT	SMI: 21% Patients with high-risk ischemia had higher event rates at follow-up compared to lesser SMI (3.1% vs. 0.4%, *p* = 0.0001)
Yamasaki et al. [39]	2010	Asymptomatic, with type 2 DM and high-risk features (485)	Stress/Rest Tc-99m tetrofosmin SPECT	SSS ≥ 9 was independently associated with a higher incidence of MACE (HR 3.39, 95% CI 1.78–6.43, *p* = 0.0002)
Xiao-Rong et al. [40]	2019	Asymptomatic, with type 2 DM (614)	Adenosine Tc-99m sestamibi SPECT	SMI: 21.3% Predictors of SMI: male sex, diabetic retinopathy, LDL-C
Patel et al. [41]	2023	DM without known CAD (2730)	Rb-82 Rest/Stress PET	SMI: 30.5% (significant in 12.5%) Reduced MBFR in 62% of asymptomatic patients CMD present in approximately 33% irrespective of symptoms Reduced MBFR and not SMI or symptoms were predictive of incident death

CAD: coronary artery disease; ETT: exercise treadmill test; SMI: silent myocardial ischemia; ACS: acute coronary syndrome; DM: diabetes mellitus; TTE: transthoracic echocardiography; LV GLS: left ventricular global longitudinal strain; OR: odds ratio; CI: confidence interval; DSE: dobutamine stress echocardiography; ESE: exercise stress echocardiography; MACCE: major adverse cardiac and cerebrovascular events; LAD-CFVR: left anterior descending artery coronary flow velocity reserve; MACE: major adverse cardiovascular events; HR: hazard ratio; DS: dobutamine stress; MRI: magnetic resonance imaging; SPECT: single-photon emission computed tomography; SSS: summed stress score; LDL-C: low-density lipoprotein cholesterol; PET: positron emission tomography; MBFR: myocardial blood flow reserve; CMD: coronary microvascular dysfunction.
